# Effective Corrosion Inhibition of Carbon Steel in Hydrochloric Acid by Dopamine-Produced Carbon Dots

**DOI:** 10.3390/polym13121923

**Published:** 2021-06-10

**Authors:** Mingjun Cui, Yue Yu, Yuxuan Zheng

**Affiliations:** 1 Key Laboratory of Impact and Safety Engineering, Ministry of Education, School of Mechanical Engineering and Mechanics, Ningbo University, Ningbo 315211, China; cuimingjun@nbu.edu.cn; 2 Key Laboratory of Marine Materials and Related Technologies, Zhejiang Key Laboratory of Marine Materials and Protective Technologies, Ningbo Institute of Materials Technology and Engineering, Chinese Academy of Sciences, Ningbo 315201, China; yuyue@nimte.ac.cn

**Keywords:** corrosion inhibition, Q235 carbon steel, NCDs, electrochemical techniques, Langmuir adsorption

## Abstract

In present study, novel nitrogen doped carbon dots (NCDs) are synthesized using a green material—dopamine—as a precursor and studied as corrosion inhibitors for Q235 carbon steel in 1 M HCl solution. According to the electrochemical results, it is found that NCDs acting as a mixed-type corrosion inhibitor can effectively retard the acid corrosion of carbon steel, and their inhibition efficiency increases with the concentration increasing from 50 to 400 ppm. The highest inhibition efficiency is 96.1% in the presence of 400 ppm NCDs at room temperature. Additionally, the adsorption of NCDs obeys the Langmuir adsorption isotherm. In addition, weight loss results show that the inhibition efficiency in the presence of 400 ppm NCDs increases with prolonged exposure time and rising temperature (298–328 K), owing to the strong adsorption of NCDs on the steel surface, and the η value is 92.2% at 60 h of immersion and 86.2%, 89.1%, 90.6% and 92.9% at 298, 308, 318 and 328 K, respectively. Surface analysis by scanning electron microscope (SEM), laser scanning confocal microscope (LSCM) and X-ray photoelectron spectroscopy (XPS) further proves the formation of a protective NCD film on the steel surface.

## 1. Introduction

The carbon steel extensively used in various industries is susceptible to severe corrosion in aggressive environments, especially in an acid medium. Hence, taking effective approaches to retard the acid corrosion of metals is of great significance. Usually, organic inhibitors including different active sites (e.g., heteroatoms (O, N, S and P), π bonds and polar functional groups) are employed to protect the steel from corrosion by forming an adsorbed film on the steel surface, which is because these active sites can promote the physical or chemical adsorption of inhibitors on the steel surface [1,2,3].

However, with the improvement in environmental awareness, most of the traditional organic inhibitors are not applicable anymore, owing to their harm to the environment and live beings. Hence, the increasing demand for safe and sustainable inhibitors has caused researchers to develop environmentally benign corrosion inhibitors. In this regard, the natural extracts of plants and fruits [4,5,6,7], ionic liquids [8,9], drugs [10,11,12] and biopolymers [13,14] have been studied as effective green corrosion inhibitors to prevent the acid corrosion of metal substrates. However, these green corrosion inhibitors are still limited in practical applications owing to the high-cost, time-consuming and tedious synthesis steps or limited shelf life.

In recent years, carbons dots, as emerging nanomaterials, have drawn much attention because of their easy preparation and superior properties [15,16,17] (such as good water solubility, low toxicity, excellent photoluminescence, etc.), and have shown great potential in a series of applications. In particular, as newcomers, carbons dots have been involved in some anticorrosive studies. In a previous study, we found that nitrogen-doped carbon dots (NCDs) prepared through a hydrothermal process of 4-aminosalicylic acid could effectively retard the acid corrosion of carbon steel at room temperature, and the inhibition efficiency was up to 91% in the presence of 10 ppm NCDs [18]. Afterwards, NCDs prepared with single source material (e.g., ammonium citrate, p-phenylenediamine and o-phenylenediamine) [19,20] or two kinds of source materials (citrate acid-ethanol amine, methacrylic acid- n-butylamine, citric acid- imidazole, citric acid- L-histidine) [21,22,23,24] were gradually reported, and they also exhibited good corrosion inhibition for metals exposed to acid solutions. Lately, the corrosion inhibition effect of double element-doped (N and S) carbon dots is starting to be studied [25]. However, owing to the difference in the source materials, the corrosion inhibition ability of carbon dots is also different. Another important thing is that the source materials used in these investigations are still toxic, and most of the investigations only focused on the effect of NCD concentration on the corrosive behavior of metals in acid solution, but ignored the inhibition effect of NCDs under various exposure times and rising temperatures.

Hence, in this investigation, a natural nontoxic material, dopamine—2-(3,4-dihydroxyphenyl)-ethylamine was selected for the preparation of NCDs. This is because self-polymerization happens easily between the dopamine molecules in common solutions to form poly(dopamine) that can adhere on a variety of materials, which would be helpful for the corrosion protection of metals. The inhibition effect for Q235 carbon steel in 1 M HCl solution was comprehensively studied in terms of NCD concentration, exposure time and temperature. The micromorphology and chemical structures for the prepared NCDs were characterized by SPM and XPS. The corrosion behavior of carbon steel in 1 M HCl solution in the presence of NCDs was researched based on electrochemical methods and weight loss measurements. Eventually, surface analysis obtained from the SEM and XPS results provided clear evidence for the adsorption of NCDs against the acid corrosion of carbon steel.

## 2. Experimental Section

### 2.1. Materials 

Dopamine hydrochloride was purchased from Aladdin. Hydrochloric acid was bought from Sinopharm Chemical Reagent Co., Ltd. (Shanghai, China). Q235 carbon steel sheets were purchased from local suppliers. The corrosive solution (1 M HCl solution) was prepared by diluting the concentrated hydrochloric acid with deionized water.

### 2.2. Preparation and Characterization of NCDs

The preparation of NCDs was realized using a hydrothermal method according to a previous report [26]. During the preparation process, a certain amount of dopamine was dissolved with deionized water in a beaker, then the mixture was transferred from the beaker into a 100 mL PTFE autoclave, and heated at 180 °C for 6 h in a drying oven. After natural cooling to room temperature, the obtained black–brown solution was dialyzed with deionized water by a dialysis bag (500 MWCO) for 24 h and the deionized water was changed every 6 h. Eventually, the NCD powder was collected by removing water through a rotary evaporation process and dried at 60 °C for 24 h. 

The micromorphology and elemental composition of NCDs were characterized by a scanning probe microscope (SPM, Dimension ICON, Bruker, Billerica, MA, United States) and X-ray photoelectron spectroscopy (XPS, Axis Ultra DLD, Kratos, UK).

### 2.3. Electrochemical Measurements

Electrochemical measurements were carried out on a CHI760E electrochemical workstation (Chenhua, Shanghai, China) with a classic three-electrode system, where Q235 carbon steel sheets (40 mm × 30 mm × 2 mm) were used as the working electrode with 1 cm^2^ of exposed area, a platinum sheet (10 mm × 10 mm) was used as the counter electrode and a saturated calomel electrode (SCE) was used as the reference electrode. Prior to the electrochemical measurements, the steel sample was first exposed to 1 M HCl solution for 6 h, and then the open circuit potential (OCP) test was carried out for 30 min to determine whether the system reached a steady state. Then, the electrochemical impedance spectroscopy (EIS) measurement was carried out at OCP in a frequency ranging from 10^−2^ Hz to 10^5^ Hz with 5 mV of amplitude. Subsequently, the potentiodynamic polarization test was performed in potential ranging from +250 mV to −250 mV with respect to OCP, accompanied with a sweep rate of 0.5 mV/s. To investigate the inhibition effect of NCDs with prolonged exposure time, EIS measurement was also performed at 12 h and 24 h of exposure in HCl solution. Zsimpwin software was used to fit the EIS data. The above measurements were conducted at room temperature. In order to ensure the reproducibility of the results, each test was performed three times. The inhibition efficiency of NCDs was calculated according to the Equations (1) and (2), where *i_corr_* is the corrosion current density and *R_p_* is the polarization resistance [27,28].

(1) $$\eta =\frac{R_{p}^{inh}-R_{p}^{HCl}}{R_{p}^{inh}}\times 100$$

(2) $$\eta =\frac{i_{corr}^{HCl}-i_{corr}^{inh}}{i_{corr}^{HCl}}\times 100$$

### 2.4. Weight Loss Measurement

According to the ASTM standard, weight loss measurement was performed to determine the corrosion rate of Q235 carbon steel in 1 M HCl solution with and without NCDs as well as the inhibition efficiency of NCDs. The pre-polished steel samples (10 mm × 10 mm × 1 mm) were first cleaned with acetone, ethanol and deionized water in turn, and then dried with high-purity nitrogen. The initial mass ($m_{0}$) of steel samples before exposure to corrosive medium was first weighed with an analytical balance and recorded. Then, the steel samples were immersed in different solutions for 60 h at 298 K, and weighed at different time intervals. To ensure the reliability of the results, the steel samples were weighed three times. Similarly, the corrosion rates of steel samples at different temperatures (308 K, 318 K and 328 K) were also measured, and the temperature of the corrosive medium was controlled by placing the sealed corrosion bottle in a temperature-constant oven. The corrosion rate ($V_{corr}$) of the steel samples in different solutions and inhibition efficiency (η) of NCDs can be calculated according to the following equations [29,30]:

(3) $$V_{corr}=\frac{m_{0}-m_{t}}{A\times t}$$

(4) $$\eta =\frac{V_{corr}^{HCl}-V_{corr}^{inh}}{V_{corr}^{HCl}}\times 100$$

where *m_0_* and *m_t_* are the mass of steel samples before exposure and after *t* h of exposure to corrosive solution, respectively. *A* is the exposed area of the steel sample in corrosive solution, *t* is the exposure time and $V_{corr}^{HCl}$ and $V_{corr}^{inh}$ correspond to the corrosion rate of steel samples in 1 M HCl solution in the absence and presence of NCDs, respectively.

### 2.5. Surface Analysis

After the weight loss measurement, the steel samples were taken out from the corrosive medium and dried in a vacuum drying oven. The surface morphology and roughness of the steel samples before and after immersion in corrosive medium were characterized by a scanning electron microscope (SEM, EVO18, ZEISS, Germany) with 20 kV of acceleration voltage and a laser scanning confocal microscope (LSCM, LSM700, ZEISS, Germany). Meanwhile, XPS was also used to check the bonding state of elements in the absence and presence of NCDs.

## 3. Results and Discussions

### 3.1. Characterization of NCDs 

**Figure 1:**
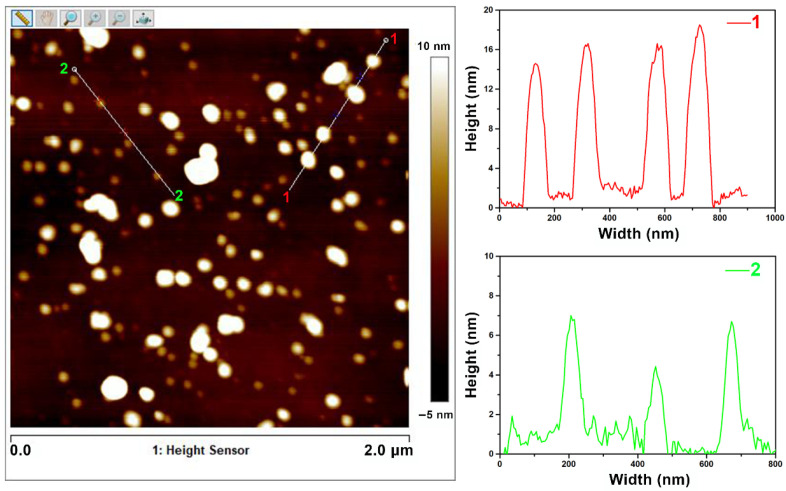
SPM micromorphology of NCDs and corresponding height profiles of lines.

**Figure 2:**
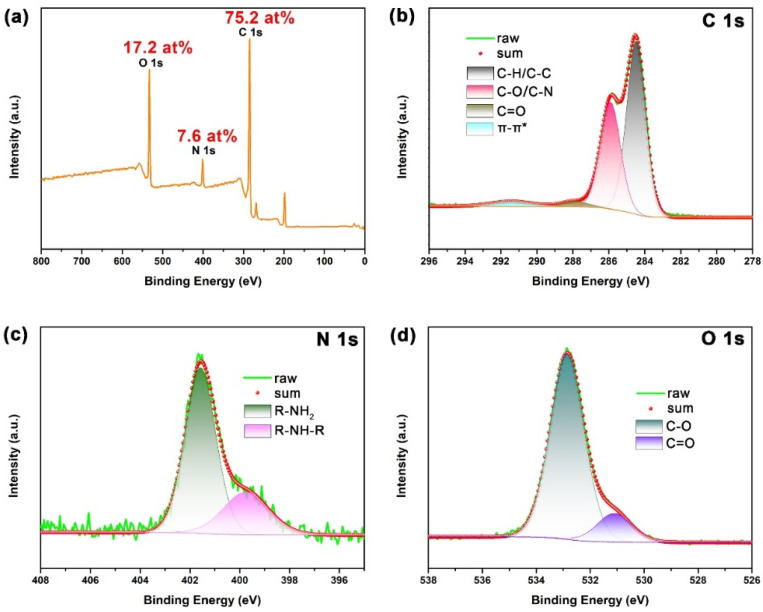
The XPS (**a**) survey spectrum of NCDs and high-resolution spectra for (**b**) C 1s, (**c**) N 1s and (**d**) O 1s.

SPM was used to examine the micromorphology of the prepared NCDs and corresponding height profiles. As shown in Figure 1, NCDs exist in the form of nanoparticles with different sizes, and the main size for the nanoparticles marked with line 1 and line 2 is 14–19 nm and 4–7 nm, respectively. Further, the elemental composition of the prepared NCDs was characterized by XPS. As presented in Figure 2a, the XPS survey spectrum of NCDs exhibits three main peaks that include C 1s at 284.0 eV, N 1s at 400.0 eV and O 1s at 532.0 eV, suggesting that NCDs mainly consist of C, N and O. Additionally, the atomic concentration for C, N and O is 75.2 at%, 7.6 at% and 17.2 at%, respectively. For the C 1s spectrum (Figure 2b), four peaks can be obtained owing to the presence of C–C/C–H at 284.6 eV, C–O/C–N at 285.9 eV, C=O at 287.9 eV and π–π* at 291.4 eV [31]. Herein, the appearance of C–O and C=O implies the presence of catechol and quinone groups in NCDs [31,32]. In case of the N 1s spectrum in Figure 2c, two peaks at 399.8 and 401.6 eV correspond to R–NH–R and R-NH_2_, respectively [26,31]. The O 1s spectrum in Figure 2d can be resolved into two peaks at 531.1 and 532.8 eV, which are ascribed to C=O and C–O, respectively [26].

### 3.2. The Effect of NCD Concentration

**Figure 3:**
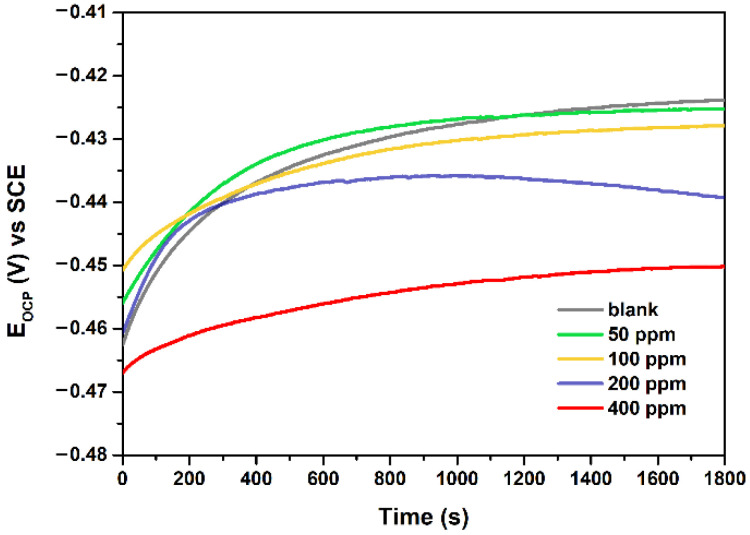
Variation of OCP with increasing time for Q235 steel in 1 M HCl solution in the absence and presence of different concentrations of NCDs.

Before the EIS tests, an OCP test was carried out for 30 min to make the system reach a steady state. Figure 3 shows the variation of OCP value with increasing immersion time for Q235 carbon steel in 1 M HCl solution in the absence and presence of different concentrations of NCDs. The E_OCP_ values for all samples increase gradually and then reach a stable value during the 30 min of the OCP test. After adding the NCDs, E_OCP_ plots exhibit a negative shift compared with the blank solution, and the OCP value varies from −0.424 V (blank solution) to −0.45 V (in the presence of 400 ppm NCDs). 

**Figure 4:**
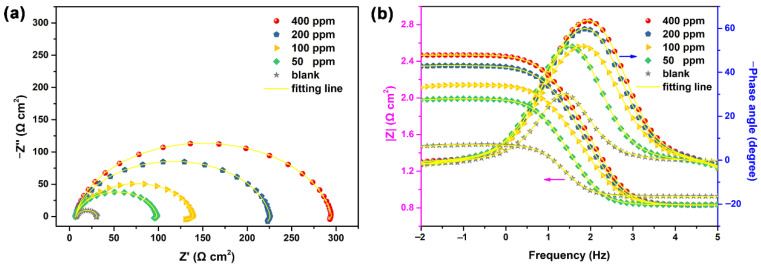
The EIS results of Q235 carbon steel in 1 M HCl solution in the absence and presence of different concentrations of NCDs, (**a**) Nyquist plots and (**b**) Bode plots.

Subsequently, the EIS technique was used to study the corrosion behavior of carbon steel in 1 M HCl solution in the absence and presence of NCDs. As presented in Figure 4a, the Nyquist plots consist of a depressed capacitive loop and an inductive loop, in which the semi-diameter of the capacitive loop increases with the increasing NCD concentration, indicating that the addition of NCDs effectively inhibits the charge transfer process at the steel–solution interface. The same depressed shape for all Nyquist plots suggests that NCDs have no effect on the corrosion process of steel in HCl solution, and this depressed shape is mainly attributed to the corrosion-induced inhomogeneity and adsorbed species on the metal surface [27,33]. In the case of the Bode plots in Figure 4b, the impedance modulus value at the lowest frequency (10^−2^ Hz) in the presence of NCDs is higher than that in blank solution, and rises with increasing NCD concentrations. The highest impedance modulus value in the presence of 400 ppm NCDs is 295 Ω cm^2^, which is 10 times higher than that in blank solution. Furthermore, the phase angle peak becomes higher and broader when NCDs are added into the HCl solution, and this phenomenon becomes more marked with increasing NCD concentration. These findings manifest the strong adsorption of NCDs on the steel surface and better corrosion inhibition ability of NCDs at higher concentrations.

**Figure 5:**
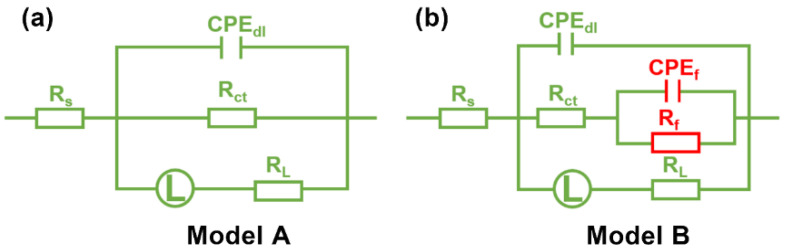
The equivalent circuit models used to fit the EIS results.

Then, the EIS results were fitted with the equivalent circuit models shown in Figure 5. For the samples exposed to blank solution and in the presence of 50 ppm of NCDs, only one time constant reflecting the charge transfer can be seen from the Bode–phase angle plots. In addition, the appearance of an inductive loop in the Nyquist plots and the negative phase angle value in the Bode plots indicate the presence of an inductance element (*L*). Thus, the equivalent circuit (Model A) is able to fit the EIS results. In Model A, *R_s_* is the solution resistance, *R_ct_* and *CPE_dl_* correspond to the resistance and double layer capacitance of the charge transfer process and *L* and *R_L_* are the inductance and resistance assigned to a slow frequency intermediate process [34]. In the case of the samples exposed to 1 M HCl with 100, 200 and 400 ppm of NCDs, the Bode–phase angle plots show higher and broader peaks owing to the strong adsorption of NCDs on the steel surface, although it is difficult to distinguish two time constants from the plots. At this time, Model A is not able to fit the EIS results of these samples. A new equivalent circuit (Model B) is proposed, in which *R_s_* is the solution resistance, *R_ct_* and *CPE_dl_* refer to the charge transfer process and R_f_ and *CPE_f_* are the resistance and capacitance of adsorbed NCD film on the steel surface. R_L_ and L are the inductance and resistance assigned to a slow frequency intermediate process, indicating the charge transfer of steel dissolution in the presence of adsorbed NCD film [34]. As shown in Figure 4, the experimental data can be well matched with the fitting lines obtained according to the proposed equivalent circuits. The electrochemical parameters obtained from the fitting process are summarized in Table 1, and the *η* value is calculated according to Equation (1). It is evident from Table 1 that the *R_ct_* value increases after the addition of NCDs with respect to that in blank solution, and this increase becomes more remarkable with the increasing NCD concentration, indicating that the adsorbed NCDs on the steel surface effectively retard the corrosion process of steel in corrosive medium. Correspondingly, the *CPE_dl_* value declines markedly with increasing NCD concentration, owing to the replacement of adsorbed water molecules on the steel surface by NCDs, indicating the increase in the electric double layer thickness at the steel–solution interface [28,30,35]. The η value of NCDs also shows a growing trend with the increasing NCD concentration, and reaches 95.2% in the presence of 400 ppm NCDs. 

**Figure 6:**
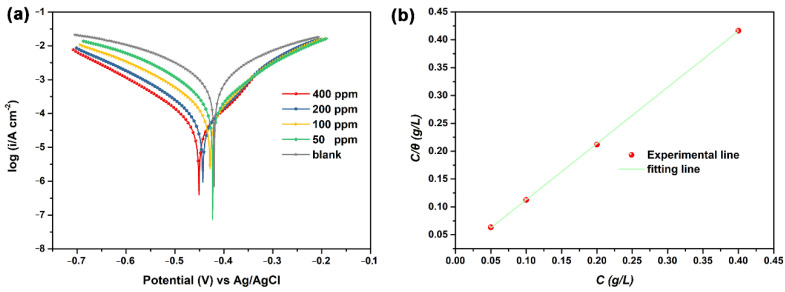
(**a**) The potentiodynamic polarization plots of Q235 carbon steel in 1 M HCl solution in the absence and presence of NCDs, and (**b**) Langmuir adsorption plot obtained from potentiodynamic polarization plots of Q235 carbon steel in the absence and presence of NCDs.

Figure 6a displays the potentiodynamic polarization plots of carbon steel in 1 M HCl solution in the absence and presence of various concentrations of NCDs, and corresponding electrochemical parameters obtained from Tafel extrapolation are shown in Table 2. It can be seen from Figure 6a that the cathodic branches of polarization plots for all samples are nearly parallel with each other, suggesting that the introduction of NCDs has no effect on the cathodic reaction of the steel [29,36]. Furthermore, as listed in Table 2, obvious negative shifts for corrosion potential (*E_corr_*) can be observed with increasing NCD concentration and the maximum displacement in *E_corr_* is 31 mV, proving that the studied NCDs act as a mixed-type inhibitor [33,37]. Meanwhile, the cathodic Tafel slope (*β_c_*) and anode Tafel slope (*β_a_*) decrease gradually with the addition of NCDs, also confirming that NCDs as a mixed inhibitor can inhibit both anodic and cathodic reactions of steel in acid solution. As a result, the corrosion current density of samples (*i_corr_*) declines dramatically from 878 μA cm^−2^ to 187 μA cm^−2^ with the first addition of 50 ppm NCDs, and continues to decline to 34.2 μA cm^−2^ with the further increase in the NCD concentration, indicating the enhancement in the corrosion inhibition efficiency of NCDs. It can be seen that the *η* value reaches up to 96.1% in the presence of 400 ppm NCDs, which is nearly consistent with the *η* value obtained from EIS results. The above results reveal that the adsorption of NCDs on the steel surface could provide excellent protection from acid corrosion of steel.

To study the interaction between NCDs and steel, the surface coverage (*θ*) obtained from the potentiodynamic polarization results are used and fitted by different adsorption isotherms (e.g., Langmuir, Freundlich, Temkin, Frumkin). Among these adsorption isotherms, the Langmuir isotherm is the most appropriate to fit our data, whose general form can be expressed as shown in Equation (5) [37,38,39]:

(5) $$\frac{C}{\theta }=C+\frac{1}{K_{ads}}$$

where *C* is the NCD concentration (g L^−1^) and *K_ads_* is the adsorption–desorption equilibrium constant. As presented in Figure 6b, the plot of *C/θ* versus *C* exhibits a linear relation and its regression coefficient is 0.9999, meaning that the adsorption of NCDs on the steel surface follows the Langmuir adsorption isotherm. In addition, the *K_ads_* value can be calculated according to the intercept of plot and has a relation to the free energy of adsorption (Δ*G_ads_*) with the following equation [30,40,41]:

(6) $$\Delta G_{ads}=-RTln\left(1000K_{ads}\right)$$

where *R* is the molar gas constant (8.314 J mol^−1^ K^−1^) and *T* is the absolute temperature (298 K). The constant value of 1000 is the water concentration in solution in g L^−1^. The *K_ads_* and Δ*G_ads_* values are 83.9 L g^−1^ and −28.1 kJ mol^−1^, respectively. The negative sign of Δ*G_ads_* and its value between −20 and −40 kJ mol^−1^ mean that the adsorption of NCDs on the steel surface is spontaneous, and involves the complex of physical (electrostatic interaction) and chemical adsorption [28]. The main mechanism is that NCDs can adsorb on the steel surface through interaction between the N active site and Fe atoms. 

### 3.3. Effect of Exposure Time and Temperature

Further, the corrosion inhibition behavior of NCDs for Q235 carbon steel in 1 M HCl solution with prolonged exposure time and rising temperature was also studied. In this section, the NCD concentration with the best inhibition efficiency in the concentration range studied above was chosen for the following investigations. 

**Figure 7:**
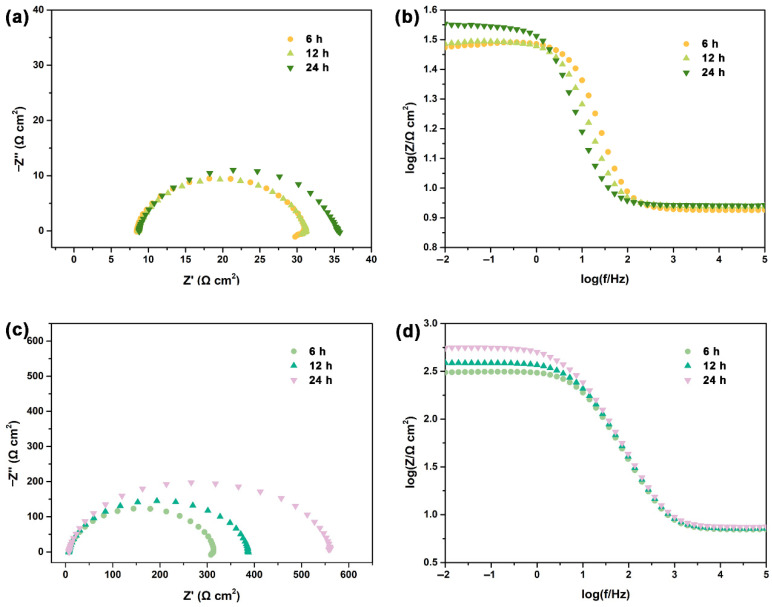
The EIS results of Q235 carbon steel after various durations of exposure to 1 M HCl solution in the (**a**,**b**) absence and (**c**,**d**) presence of 400 ppm NCDs.

Figure 7 presents the Nyquist and Bode plots for the steel after various durations of exposure to 1 M HCl solution in the absence and presence of 400 ppm NCDs. In the blank solution (Figure 7a,b), the semi-diameter of the Nyquist plots varies slightly during 24 h of exposure, and the impedance modulus is about 32 Ω cm^2^. In the presence of 400 ppm NCDs (Figure 7c,d), the semi-diameter of Nyquist plots increases markedly as the exposure time is prolonged, and the impedance modulus of the samples increases from 309 to 562 Ω cm^2^, implying the continuous coverage of the steel surface by adsorbed NCDs. In this case, the corrosion of the steel only occurs on the uncovered steel surface or the pores of adsorbed film.

**Figure 8:**
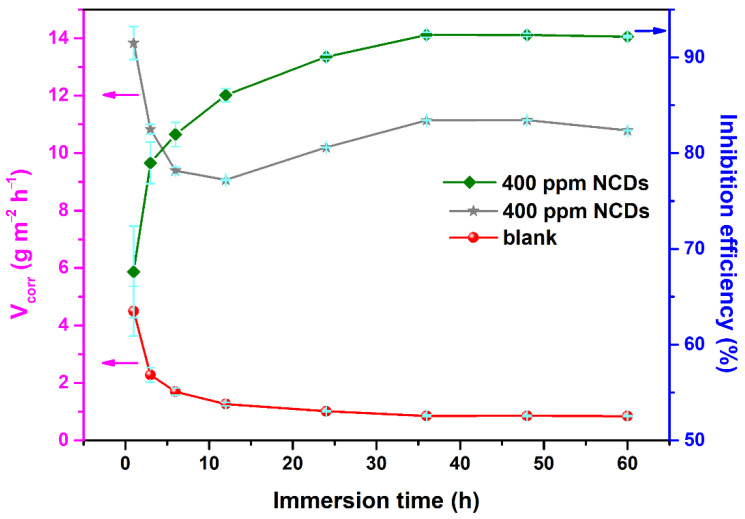
Evolution of corrosion rate and inhibition efficiency with prolonged exposure time in the absence and presence of 400 ppm NCDs (the error is the standard error).

Figure 8 presents the weight loss results of Q235 carbon steel in 1 M HCl solution in the absence and presence of 400 ppm NCDs. In the first 12 h of immersion, the corrosion rates for all samples decrease and inhibition efficiency increases with the extension of exposure time, implying the continuous adsorption of NCDs on the steel surface. As the immersion time is further prolonged, the corrosion rate and inhibition efficiency almost reach a steady value. As seen from Figure 8, the corrosion rate after 60 h of immersion in blank solution is about 10.8 g m^−2^ h^−1^, which is nearly 13 times larger than that in the presence of 400 ppm NCDs (0.84 g m^−2^ h^−1^). Additionally, the highest inhibition efficiency in the presence of 400 ppm NCDs is approximately 92.2%, revealing the superior corrosion inhibition of NCDs by adsorption on the steel surface.

**Figure 9:**
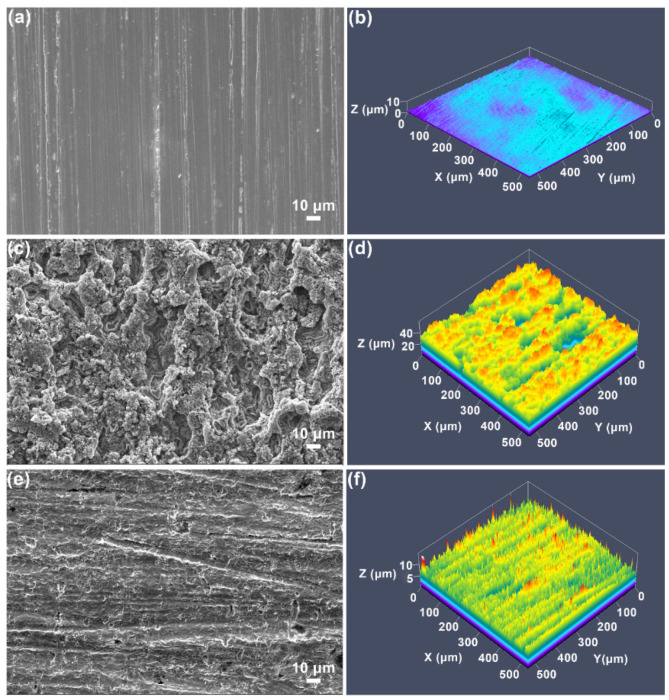
SEM and LSCM images of Q235 carbon steel surface before and after 60 h of exposure to 1 M HCl solution in the absence and presence of 400 ppm NCDs, (**a**,**b**) polished steel, (**c**,**d**) in blank solution, (**e**,**f**) in the presence of 400 ppm NCDs.

After 60 h of exposure, the surface morphology and roughness of the samples were checked by SEM and LSCM, respectively. Prior to the exposure to corrosive medium, the steel surface was smooth and just showed polished scratches (Figure 9a). The surface roughness of the steel was about 0.27 μm (Figure 9b). After 60 h of exposure to 1 M HCl solution, the steel surface was severely corroded and became loose and porous (Figure 9c). Compared to the steel without corrosion, its surface roughness increased dramatically to 5.18 μm (Figure 9d). In contrast, after the addition of 400 ppm NCDs, the corrosion of the steel surface was significantly decreased, and the polished scratches were still visible (Figure 9e). At this moment, the surface roughness of the corroded steel is only approximately 1.0 μm (Figure 9f). These results indicate that the adsorbed NCDs effectively retard the acid corrosion of steel.

Further, the effect of temperature on the adsorption behavior of NCDs on the steel surface was also studied by the weight loss measurement. The weight loss results of samples exposed to these two solutions at four temperatures (298, 308, 318 and 328 K) are listed in Table 3. It is noted that both corrosion rate and inhibition efficiency in the absence and presence of 400 ppm NCDs increase with the rising temperature. This is because the corrosion reaction of steel is always accelerated by elevating temperature, thus leading to a higher corrosion rate. The change in the inhibition efficiency suggests that the rising temperature promotes the chemical adsorption of NCDs on the steel surface [37,42]. 

**Figure 10:**
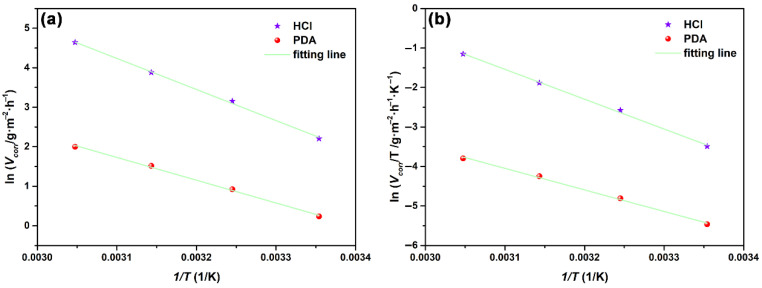
The plots of (**a**) ln*V_corr_* versus 1/*T* and (**b**) $\ln\frac{V_{corr}}{T}$ versus 1/*T* for steel exposed to 1 M HCl in the absence and presence of 400 ppm NCDs.

To further understand the adsorption of NCDs on the steel surface, the apparent activation energy (*E_a_*) for the corrosion process of steel in the absence and presence of 400 ppm NCDs was calculated according to the Arrhenius equation [37,42]:

(7) $$\ln V_{corr}=\frac{-E_{a}}{RT}+lnA$$

where *V_corr_* is the corrosion rate of steel in corrosive medium and *A* is the Arrhenius pre-exponential factor. It can be seen from Figure 10a that ln*V_corr_* versus 1/*T* shows a linear relation. The *E_a_* value can be calculated from the slope of the plot and is listed in Table 4. The *E_a_* value in the presence of 400 ppm NCDs is lower than that in blank solution, suggesting that the adsorption of NCDs on the steel surface is dominated by chemisorption with rising temperature [36,43]. In addition, an alternative Arrhenius equation is used to calculate the entropy of activation (Δ*S_0_*) and enthalpy of activation (Δ*H_0_*) [37,42].

(8) $$\ln\frac{V_{corr}}{T}=\frac{-\Delta H_{0}}{RT}+ln\frac{R}{Nh}+\frac{\Delta S_{0}}{R}$$

Herein, N and h are Avogadro’s number (6.02 × 10^23^) and Planck’s constant (6.626 × 10^−34^ J·s), respectively. Figure 10b presents the plots of $\ln\frac{V_{corr}}{T}$ versus 1/*T* in the absence and presence of 400 ppm NCDs. The value of ΔH_0_ and ΔS_0_ can be obtained from the slope (−ΔH_0_/R) and intercept (ln(R/Nh) + ΔS_0_/R) of the plots. As shown in Table 4, the ΔH_0_ value in the absence and presence of 400 ppm NCDs is 62.79 and 45.34 kJ mol^−1^, respectively, and its positive sign confirms that the corrosion of steel in HCl solution is an endothermic process [44]. The ΔS_0_ shows a negative shift from −15.71 J mol^−1^ K^−1^ in blank solution to −90.62 J mol^−1^ K^−1^ in the presence of 400 ppm NCDs, illustrating the increase in the degree of order for the adsorbed NCDs on the steel surface [37,44].

### 3.4. XPS Analysis on the Corroded Surface

**Figure 11:**
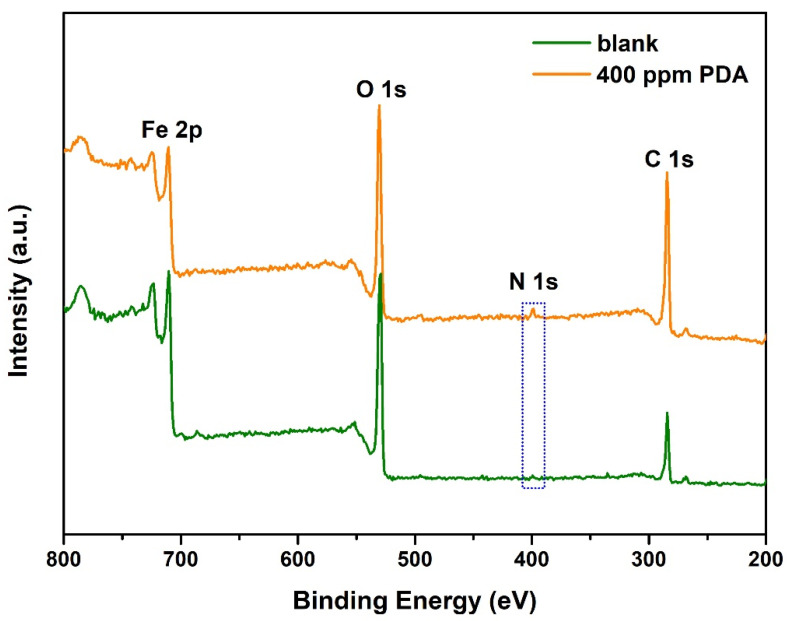
XPS surveys spectra for the Q235 carbon steel experiencing 60 h of exposure to 1 M HCl solution in the absence and presence of 400 ppm NCDs.

**Figure 12:**
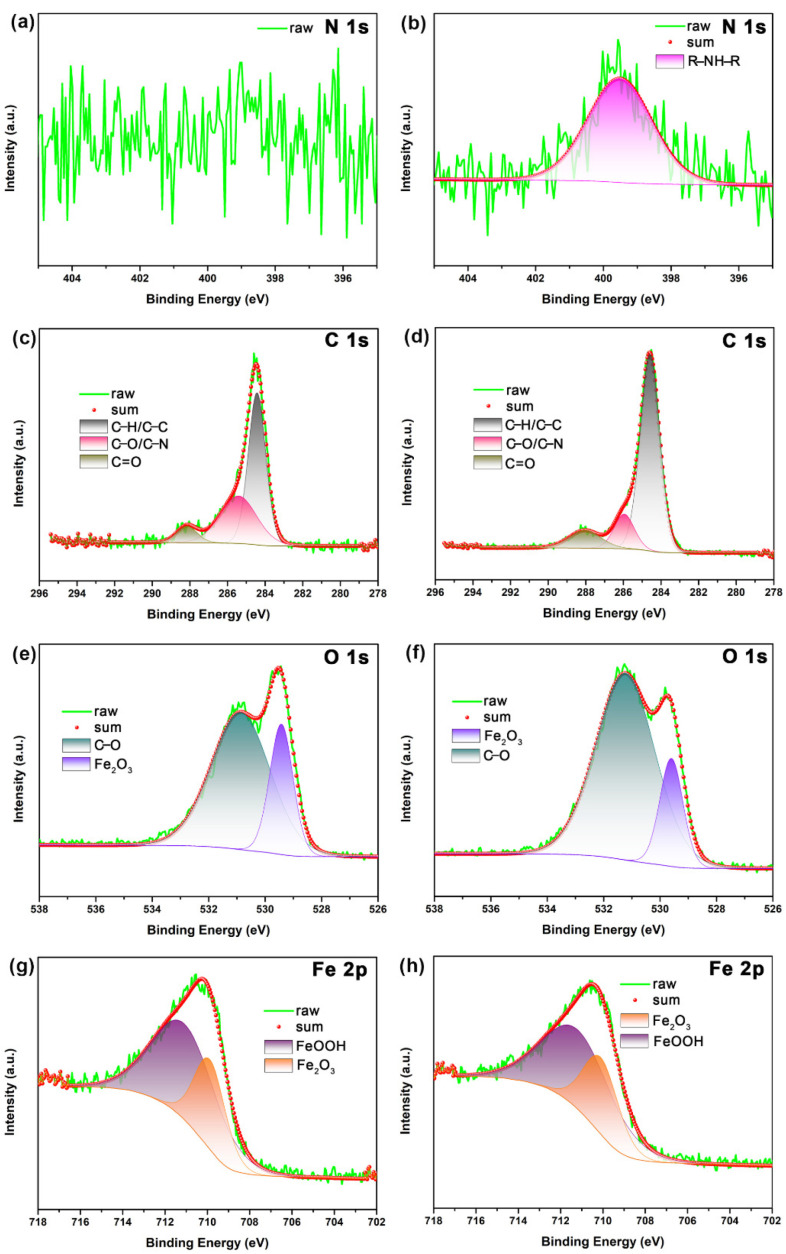
The high-resolution XPS spectra of the steel surface after exposure to 1 M HCl solution and in the presence of 400 ppm NCDs, (**a**,**b**) N 1s, (**c**,**d**) C1s, (**e**,**f**) O 1s and (**g**,**h**) Fe 2p.

The XPS technique was carried out to verify the adsorption of NCDs on the steel surface and evaluate the bonding information of NCDs on the steel surface. For the sake of contrast, the XPS spectra for the steel in the absence and presence of NCDs were investigated. Figure 11 presents the XPS survey spectra of the steel surface after exposure to 1 M HCl solution in the absence and presence of 400 ppm NCDs. With respect to the XPS survey spectrum in blank solution, the appearance of the N peak and the increasing intensity of the C 1s peak in the presence of NCDs indicate the adsorption of NCDs on the steel surface. It can be seen from Table 5 that the content of N and C increases from 0.2% and 20.39% to 2.89% and 34.61%, respectively. Then, the high-resolution spectra for different elements were further analyzed, as shown in Figure 12. Distinctly, the N peak is difficult to detect after exposure to blank solution while an obvious N peak at 399.6 eV can be observed and assigned to R-NH-R, proving the existence of NCDs on the steel surface (Figure 12a,b). For the remaining elements, their high-resolution spectra in the absence and presence of 400 ppm NCDs are nearly consistent. In the case of C 1s spectra (Figure 12c,d), three peaks can be seen at 284.6, 286.0 and 288.1 eV, corresponding to C–H/C–C, C–O/C–N and C=O, respectively [31]. The O 1s spectra in Figure 12e and f are composed of two peaks, in which the peak at 529.6 eV is associated with the presence of Fe_2_O_3_ [10], and another peak at 531.2 eV represents C=O [26]. By comparison, the area ratio of C–O to Fe_2_O_3_ in the presence of NCDs is higher than that in blank solution, indicating the coverage of the steel surface by adsorbed NCDs. For Fe 2p spectra (Figure 12g,h), two peaks corresponding to Fe_2_O_3_ and FeOOH can be obtained at 710.3 and 711.6 eV, respectively [25]. 

## 4. Conclusions

In this investigation, NCDs were successively prepared by a hydrothermal process of dopamine, and corresponding corrosion inhibition behavior of NCDs on the Q235 carbon steel in 1 M HCl solution was systematically studied in terms of the NCD concentration, exposure time and temperature. As confirmed by electrochemical and weight loss results, NCDs acting as a mixed-type corrosion inhibitor could effectively inhibit the acid corrosion of Q235 carbon steel. The inhibition efficiency of NCDs increased with the increasing NCD concentrations, and the highest inhibition efficiency of up to 96.1% was obtained in the presence of 400 ppm NCDs. With increasing immersion time, the inhibition efficiency of NCDs increased gradually and finally reached a steady value. In addition, the inhibition efficiency was also found to increase proportionally from 86.15% to 92.88% with the rising temperature (298–328 K). The mechanism is that NCDs can absorb on the steel surface to prevent the corrosion of steel, and the adsorption of NCDs on the steel surface follows the Langmuir adsorption model. Further surface analysis found increasing C and N content on the steel surface in the presence of NCDs, indirectly implying the adsorption of NCDs on the steel surface.

## Figures and Tables

**Table 1:** Electrochemical parameters obtained by fitting the EIS results with equivalent circuits.

	*R_s_* (Ω cm^2^)	*R_ct_* (Ω cm^2^)	*CPE_dl_*		*L* (H cm^2^)	*R_L_* (Ω cm^2^)	*CPE_f_*		*R_f_* (Ω cm^2^)	*η* (%)
			*Y_0_* × 10^5^ (Ω ^−1^cm^−2^ s^n^)	n			*Y_0_* × 10^5^ (Ω^−1^ cm^−2^ s^n^)	*n*		
blank	7.074	13.52	92.29	0.8852	105.2	3.689	/	/	/	/
50	6.781	86.65	38.38	0.8904	53.69	5.044	/	/	/	84.4
100	6.725	124.3	24.79	0.8341	63.29	9.225	/	/	/	89.1
200	6.743	162.3	11.85	0.8914	816.7	22.5	91.2	0.9044	34.48	93.1
400	6.785	221.9	8.131	0.912	74.32	6.52	65.69	0.8152	60.43	95.2

**Table 2:** Electrochemical parameters obtained from the potentiodynamic polarization plots.

	*E_corr_* (mV)	*i_corr_* (μA cm^−2^)	−*β_c_* (mV/dec)	*β_a_* (mV/dec)	*R_p_* (Ω cm^2^)	*θ*	*η* (%)
blank	−420	878	142.2	112.6	31	/	/
50	−423	187	94.4	93.5	109	0.787	78.7
100	−428	98.6	91.7	75.1	182	0.888	88.8
200	−443	49.7	76.3	70.4	320	0.943	94.3
400	−451	34.2	67.3	73.8	448	0.961	96.1

**Table 3:** The weight loss results for steel after 12 h of exposure to 1 M HCl solution in the absence and presence of 400 ppm NCDs at various temperatures.

*T* (K)	*C* (ppm)	*V_corr_* (g·m^−2^·h^−1^)	*η*
298.15	blank	9.1	/
	400	1.26	86.2
308.15	blank	23	/
	400	2.5	89.1
318.15	blank	48	/
	400	4.5	90.6
328.15	blank	104	/
	400	7.4	92.9

**Table 4:** The activation parameters for Q235 carbon steel in 1 M HCl in the absence and presence of 400 ppm NCDs.

*C* (ppm)	*E_a_* (kJ·mol^−1^)	Δ*H^0^* (kJ·mol^−1^)	Δ*S^0^* (J·K^−1^·mol^−1^)
Blank	65.38	62.79	−15.71
400	47.94	45.34	−90.62

**Table 5:** The variation of elemental composition of the steel surface after 60 h of exposure to 1 M HCl solution in the absence and presence of 400 ppm NCDs.

Elemental Composition (wt%)	Fe 2p	O 1s	N 1s	C 1s
1 M HCl	50.74	28.66	0.2	20.39
400 ppm PDA	36.87	25.62	2.89	34.61

## Data Availability

The data presented in this study are available on request from the corresponding author.
